# Survey of *Plasmodium falciparum* multidrug resistance-1 and chloroquine resistance transporter alleles in Haiti

**DOI:** 10.1186/1475-2875-12-426

**Published:** 2013-11-19

**Authors:** Maha A ElBadry, Alexandre Existe, Yves S Victor, Gladys Memnon, Mark Fukuda, John B Dame, Charles A Yowell, Bernard A Okech

**Affiliations:** 1 Department of Environmental and Global Health, University of Florida, Gainesville, FL 32610, USA; 2 National Public Health Laboratory, Ministry of Public Health and Population, Port au Prince, Haiti; 3 Blanchard Clinic, Family Health Ministries Haiti, Terre Noire, Port au Prince, Haiti; 4 Hospital Saint Croix, Leogane, Haiti; 5 Armed Forces Health Sciences Surveillance Center, Silver Spring, MD, USA; 6 Department of Infectious Diseases and Pathology, University of Florida Gainesville, Gainesville, FL 32610, USA; 7 Emerging Pathogens Institute, University of Florida, Gainesville, FL 32610, USA

**Keywords:** Hispaniola, Chloroquine, Mefloquine, Anti-malarial drug resistance, Genotyping

## Abstract

**Background:**

In Haiti where chloroquine (CQ) is widely used for malaria treatment, reports of resistance are scarce. However, recent identification of CQ resistance genotypes in one site is suggestive of an emerging problem. Additional studies are needed to evaluate genetic mutations associated with CQ resistance, especially in the *Plasmodium falciparum* multi-drug resistance*-*1 gene (*pfmdr*1) while expanding the already available information on *P. falciparum* CQ transporter gene (*pfcrt*) in Haiti.

**Methods:**

Blood samples were collected on Whatman filter cards (FTA) from eight clinics spread across Haiti. Following the confirmation of *P. falciparum* in the samples, PCR protocols were used to amplify regions of *pfmdr*1and *pfcrt* codons of interest, (86, 184, 1034, 1042, and 1246) and (72-76), respectively. Sequencing and site-specific restriction enzyme digestions were used to analyse these DNA fragments for the presence of single nucleotide polymorphisms (SNPs) known to confer resistance to anti-malarial drugs.

**Results:**

*P. falciparum* infection was confirmed in160 samples by amplifying a segment of the *P. falciparum* 18S small subunit ribosomal RNA gene (*pfssurrn*a). The sequence of *pfmdr1* in 54 of these samples was determined between codons 86,184 codons 1034, 1042 and 1246. No sequence differences from that of the NF54 clone 3D7 were found among the 54 samples except at codon 184, where a non-silent mutation was found in all samples predicted to alter the amino acid sequence replacing tyrosine with phenylalanine (Y184F). This altered sequence was also confirmed by restriction enzyme digestion. The sequence of *pfmdr*1 at codons 86, 184, 1034 and 1042 encoded the N*F*SN haplotype. The sequence of *pfcrt* codons 72-76 from 79 samples was determined and found to encode CVMNK, consistent with a CQ sensitive genotype.

**Conclusion:**

The presence of the Y184F mutation in p*fmdr1* of *P. falciparum* parasites in Haiti may have implications for resistance to antimalarial drugs. The absence of mutation in *pfcrt* at codon 76 among 79 isolates tested suggests that sensitivity to CQ in Haiti remains common. Wide-spread screening of the *pfmdr1* and *pfcrt* especially among patients experiencing treatment failure may be a useful tool in early detection of the emergence of antimalarial drug resistance in Haiti.

## Background

An estimated 30,000 cases of malaria infections are reported annually in Haiti [1,2], where transmission is hypo-endemic with sporadic epidemics fueled by heavy rainfall. Malaria infections in Haiti are dominantly caused by *Plasmodium falciparum*[3,4]. Chloroquine (CQ) has been in use as an anti-malarial since the 1950s [5], and is currently used extensively in the treatment of malaria in Haiti [1]. Recently, a national malaria treatment policy revision added single dose primaquine to target gametocytes [1]. Despite long-term use, there are scarce reports of CQ-resistant *P. falciparum* malaria in Haiti [6-8]. However, the recent reports of CQ-resistant *P. falciparum* parasites harbouring known resistant alleles of *pfcrt*[9,10] are alarming and could present serious challenges to clinical management of malaria in Haiti and diminish prospects for its elimination from Hispaniola (the Caribbean island where both Haiti and the Dominican Republic are located).

Research studies conducted more than three decades ago found only CQ sensitive *P. falciparum* strains [6,7], but very few follow-up studies have been conducted during the intervening years. In the recent past, a few studies identified the CQ-resistance-associated K76T mutation in the *pfcrt* at a very low prevalence in samples from the Artibonite valley, from Leogane, and from travellers returning from Haiti [8-10]. The potential role of resistant alleles of *pfmdr1* in CQ resistance in Haiti has yet to be investigated. In addition, there is a need to screen for the CQ resistance-associated *pfcrt* and *pfmdr1* alleles in other parts of Haiti.

Single nucleotide polymorphisms (SNPs) in the *pfmdr1* have been associated with resistance to CQ and other anti-malarial drugs including amodiaquine (AQ) and artemether-lumefantrine (AL). The *P. falciparum* glycoprotein homologue-1 found in the digestive vacuole of the malaria parasites is encoded by *pfmdr1* gene [11]. CQ diffuses to parasite food vacuole (FV) membrane neutrally where it protonates by acids present, which prevents free diffusion out the vacuole and results in accumulation of CQ in sensitive parasites. In CQ resistant parasites, changes in FV membrane proteins encoded by *pfcrt* and *pfmdr1*genes are thought to play a role in reducing accumulation of drug inside the vacuole [11-14]. The role played by *pfmdr1* mutations (N86Y, Y184F, S1034C and D1246Y) in mediating *in vivo* and *in vitro* CQ resistance has received a lot of research interest [13,15-23]. No previous reports have described alleles of *pfmdr1* that may be associated with anti-malarial drug resistance in Haiti. Therefore, this research study evaluated in Haiti the mutations in *pfmdr1* that in other regions of the world are associated with resistance to CQ and expanded the surveillance locations in Haiti for CQ resistance mutations in *pfcrt*. This study complements the anti-malarial drug resistance surveillance efforts of the Haitian government.

## Methods

### Study sites

**Figure 1:**
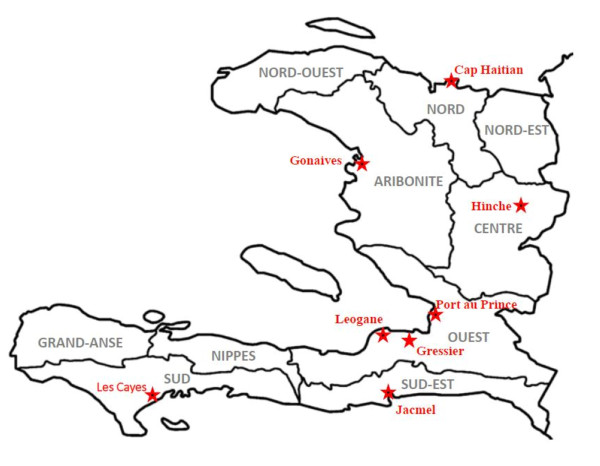
Map of Haiti showing sampling sites.

The sites where the samples originated are located in Port-au-Prince, Artibonite, North Cap Haitian, Leogane, Hinche and Jacmel (Figure 1). All sites are rural communities except the Port-au-Prince site, which is near the international airport. All locations suffer drastically from poor infrastructural facilities, which is common in Haiti. The average temperature in these sites ranges from (22°C-35°C) all year round [24] which favors malaria vector breeding and consequently malaria transmission. Peak rainfall occurs between November and January [25], and the majority of malaria cases were sampled during this period.

### Sample collection

*Plasmodium falciparum* malaria infected blood samples were collected by finger prick and preserved as dried blood spots (DBS) on FTA cards (Whatman GE Healthcare, MA, USA) between May 2010 and June 2012. Samples were obtained from patients attending study clinics, meeting the inclusion criteria (any patient above the age of two year old with malaria-like symptoms) who tested positive with rapid diagnostic test (CareStat™First Response Malaria Ag pLDH/HRP2 Combo card, Premier Medical Corporation, India) and microscopy for *P. falciparum* and agreed to sign a consent form. All patients received treatment according to the Haitian approved standard of care available at the clinics. The Haiti Ethical Review Board, University of Florida Institutional Review Board and the Office of Research Protections, US Army Medical Research and Material Command approved this study.

### DNA extraction

*Plasmodium falciparum* DNA was extracted from the DBS with a methanol extraction wash protocol as previously described [26,27]. A single disc 3 mm in diameter was punched from the DBS and placed in a 0.2 ml micro-centrifuge tube. A volume of 100 μl of absolute methanol was added and the tube incubated at room temperature for 15 min. Methanol was then decanted and the 3 mm disc left to air dry for 30 min. Sixty-five microlitres of sterile DNA-free grade water was then added to the dried disc, agitated slightly and then heated to 97°C for 30 min. The tube was then centrifuged at low speed and the supernatant stored at −20°C for use in the PCR amplification protocol.

### Detection of *Plasmodium falciparum* and amplification of *pfmdr1* by PCR

All samples were PCR tested to confirm the presence of *P. falciparum* by amplifying the small subunit ribosomal RNA (*pfssurrna*) gene [GenBank: KC906718.1] of *P. falciparum* with a one-step PCR as previously described [10]. Confirmed *P. falciparum* positive samples were subsequently used for amplification of segments of *pfmdr1* encoding five codons of interest; 86, 184, 1034, 1042 and 1246 using a nested PCR protocol as previously described [28]. All primer sequences used to amplify segments of *pfmdr1* are listed in Table 1. All PCR reactions included the following reagents: 1x Taq PCR Master Mix, 0.2 mM dNTPs, 0.75 mM MgCl_2,_ and 0.2 μM of each primer. The temperature protocol used for each reaction were as follows: one cycle at 94°C for 2 minutes, an amplification for 35 cycles (94°C for 30 sec, 45°C for 1 min, and 72°C for 1 min) and a final extension at 72°C for 5 min. Two microlitres of the product of the initial PCR was used as a template for the nested second PCR reaction in all regions. The products of the nested PCR were electrophoresed in 2% agarose gels, stained with ethidium bromide (EtBr) and visualized with a BioRad Transluminator (Universal Hood II). Amplification products were used in restriction digest analysis and sequenced as described below.

**Table 1:** **The PCR amplification primers list for *pfmr1 *** **and *pfcrt *** **used in the study**

**Gene**	**PCR step**	**Primer**	**Sequence**	**Nucleotide location**
*pfmdr1*	Nest 1	dr2	5-AGATGGTAACCTCAGTAT-3	27…44
		dr3	5-AGTCTTTTCTCCACAATA-3	756…739
	Nest 2	A4	5-TGTTGAAAGATGGGTAAAGAGCAGAAAGAG-3	24…54
		A2	5-GTCAAACGTGCATTTTTTATTAATGACCAttTA-3	584…552
	Nest 1	dr5	5-GAAATGTTTAAAGATCCAAG-3	2929…2949
		dr6	5-CAGCAAACTTACTAACAC-3	3864…3848
	Nest 2	1034f	5-AGAATTATTGTAAATGCAGCTTTATGGGGAcTC-3	3067…3099
		1042r	5-AATGGATAATATTTCTCAAATGATAAcTTAGCA-3	3299…3267
		1246f	5-ATGATCACATTATATTAAAAAATGATATGACAAAT-3	3545…3589
*pfcrt*		Pfcrt F1	5-CCGTTAATAATAAATACACGCAG-3	25…47
		Pfcrt R2	5-ATGTTTTATATTGGTAGGTGGAAT-3	694…716

### Restriction enzyme digestion for *pfmdr1* mutations

The *pfmdr1* amplification products were subjected to restriction enzyme digestion. The restriction enzymes used included Apo I/Afl III, Dra I, Dde I, AseI and EcoRV for analysis of sequences at codons 86, 184, 1034, 1042 and 1246, respectively. All enzymes were purchased from New England Biolabs (Ipswich, MA, USA). Technique was performed as previously described [28,29]. Digestion was carried out according to manufacturer instructions. The product of digestion was resolved on a 2.5% agarose gel and visualized with BioRad Transilluminator (Universal Hood II).

### Amplification of *pfcrt*

Samples positive for *pfssurrna* were used to amplify codons 72–76 of *pfcrt* gene as previously described [10,30]. A one-step PCR was carried out using Phusion flash high fidelity master mix from (New England Biolabs, Ipswich, MA,USA) with a 25 μl final volume of PCR reaction composed of 1x PCR Master Mix containing 0.2 mM dNTPs, 0.5 μM each primer, 2.5 mM MgCl2, and 1 μl of DNA extract. The cycling temperature protocol used was: two cycles at 95°C for 5 min, amplification for 40 cycles (94°C for 30 sec, 60°C for 30 sec, and 72°C for 30 sec) and a final extension at 72°C for 10 min. Products of amplification were then electrophoresed on 2% agarose gel, stained with EtBr and visualized with BioRad Transilluminator (Universal Hood II).

### Sequencing and alignment

Amplified segments of *pfmdr1* and *pfcrt* were sequenced by the Sanger chain-termination method to determine polymorphic sites associated with CQ or other anti-malarial resistance. Sequencing was done on a Biosystem 3730 Genetic Analyzer at the University of Florida’s Interdisciplinary Center for Biotechnology Research DNA Sequencing Core Laboratory. All sequences results were aligned to *P. falciparum* 3D7 *pfmdr1*and *pfcrt* sequences. [GenBank: NC_004326.1 and NC_004328.2] respectively.

## Results

A total of 375 microscopy-confirmed positive samples for *P. falciparum* were collected for analysis by PCR. A segment of the *pfssurrna* gene was successfully amplified from160 of these samples. One or more segments of interest from the *pfmdr1* gene were successfully amplified for sequence analysis from 54 samples. No mutations were detected in codons 86, 1034, 1042 and 1246 among the samples in which codons were successfully amplified. However, mutations were found at codon 184 (Y → F) in all the samples in which the codon successfully amplified which was further confirmed by restriction enzyme digestion. The number of samples that successfully amplified for each codon per site is shown in Table 2. Thirty-eight samples that amplified at codons 86, 184, 1034 and 1042 were all NFSN haplotype. In the analysis of *pfcrt*, the polymorphic region associated with CQ resistance alleles, encompassing codons 72–76, was successfully amplified from79 samples. No mutations were detected with all samples expressing the wild type sequence CVMNK. The intersection of samples from which both alleles–*pfcrt* and *pfmdr1*- successfully amplified were 50.

**Table 2:** **Presents summary of PCR amplification results and geographic distribution of samples collected in the study, number of samples amplified for different genes *pfssurrna*, *pfcrt*, and differential data on no. of samples amplified for different codons of *pfmdr1***

**Site**	**No. of samples positive for microscopy**	**Positive for *pfssurrna***	**Positive for *pfcrt***	**Codon 86-184**	**Codon 1034-1042**	**Codon 1246**	**No. of samples amplified for codons 86-1042**
Port-au-Prince	251	70	39	29	27	8	23
Leogane	40	39	17	8	11	1	4
Cap Haitian	10	2	3	0	1	0	0
L’Cay Jacmel	17	16	15	13	9	0	8
Artibonite	50	26	3	2	2	0	1
Nippes	7	7	2	2	3	0	2
Total	375	160	79	54	53	9	38

## Discussion

Although this study was limited by small sample size, it provides preliminary information about the *pfmdr1* alleles in Haiti and adds additional data on *pfcrt* alleles in parasites from both previously sampled regions of Haiti and from regions not previously sampled. This study is the first to examine in Haiti the *pfmdr1* genetic mutations linked to anti-malarial drug resistance in other regions of the world. This study found neither the K76T mutation in *pfcrt* nor the N86Y, S1034C, N1042D or D1246Y mutations in *pfmdr1*, that have been reported to confer resistance to CQ [22,23,31-33]. The absence of mutations at codons 1034 and 1042 in *pfmdr1* suggests that the *P. falciparum* parasites analysed in this study likely remain sensitive to mefloquine [34] and AL [35]. Interestingly, all the samples tested had a Y− > F mutation in codon 184 in *pfmdr1*. The widespread occurrence of this single mutation in *pfmdr1* is not unique in the literature [36,37]. Previous studies of clinical isolates of *P. falciparum* in Cambodia reported a similar widespread occurrence of single point mutations, and the mutation resulting in the Y184F mutation was predominant over other *pfmdr1* mutations [38].

Although the Y184F mutation in *pfmdr1* has been associated mainly with low level resistance to AL [39], there is no consensus in the literature for its role in CQ resistance [28,34,40]. In a previous study [15], the Y184F polymorphism was shown to play a role in CQ resistance in close association with the K76T mutation in *pfcrt*. The Y184F mutation is thought to alter the kinetics of anti-malarial drug transport [32,41], and thus efficacy. The absence of the K76T mutations in the study sample indicates that the Y184F mutation in *pfmdr1* is rarely found in Haiti in combination with K76T in *pfcrt*. Although this *pfmdr1* allele may not alone increase CQ resistance, it’s high prevalence could have an impact if the frequency of the *pfcrt* K76T allele were to increase in Haiti [8]. Other studies have shown a modification of resistance to AL in *P. falciparum* with the Y184F mutation under a N86Y and D1246Y background [36]. The absence of N86Y, S1034C, N1042D or D1246Y in *pfmdr1* alleles in this study sustains CQ or AL sensitivity in Haiti. Therefore, the findings would seem to indicate that the presence of the Y184F mutation alone would not contribute greatly to anti-malarial resistance in Haiti in the absence of wide spread K76T mutations in *pfcrt* or additional mutations in *pfmdr1*. Routine molecular surveillance of the anti-malarial drug resistance genes in Haiti combined with *in vitro* drug sensitivity studies are needed to ascertain the sensitivity of Haitian *P. falciparum* strains to the major anti-malarial medications in use in Haiti and other parts of world.

The recent reports of CQ resistance genotypes in Haiti are alarming for public health officials in Haiti and concerted efforts are needed to monitor the anti-malaria drug resistance genotypes in Haiti. The results of this study have provided background data on *pfmdr1*, which may be referenced in discussions of future *P. falciparum* anti-malarial resistance patterns in Haiti. This study also provides additional data on the prevalence of the *pfcrt* K76T allele in Haiti, but additional studies are needed to increase the sample size and provide an accurate estimate for K76T allele prevalence and distribution. Nonetheless, the findings in this study have increased the knowledge of two important anti-malaria drug resistance markers in Haiti, where widespread drug resistance has not yet been observed.

### Study limitations

The small sample size is indeed the largest limitation of this study, however due to the difficulty of the study initiation; proper sample storage for the FTA cards was lacking which affected the quality of DNA. The study lack adequate sample distribution, were some sites failed to enroll sufficient number of patients, this can be attributed to change in site administration which affected the study enrollment.

## Competing interests

The authors declare they have no competing interest.

## Authors’ contributions

MAE generated the molecular data and interpretation, contributed to writing of the manuscript. AE organized sample collection in Haiti. YV organized sample collection at Blanchard clinic. GM organized sample collection in Hospital Saint Croix. MF reviewed data and writing of manuscript. JD participated in writing manuscript. CY worked on genotyping of samples. BO designed the study, drafted manuscript, coordinated the sample collection, and data analysis. All authors read and approved the final manuscript.
